# A Western Dietary Pattern Increases Prostate Cancer Risk: A Systematic Review and Meta-Analysis

**DOI:** 10.3390/nu8100626

**Published:** 2016-10-12

**Authors:** Roberto Fabiani, Liliana Minelli, Gaia Bertarelli, Silvia Bacci

**Affiliations:** 1 Department of Chemistry, Biology and Biotechnology, University of Perugia, Perugia 06123, Italy; 2 Department of Experimental Medicine, University of Perugia, Perugia 06123, Italy; liliana.minelli@unipg.it; 3 Department of Economics, University of Perugia, Perugia 06123, Italy; gaiabertarelli@gmail.com (G.B.); silvia.bacci@unipg.it (S.B.)

**Keywords:** dietary pattern, prostate cancer, systematic review, meta-analysis

## Abstract

Dietary patterns were recently applied to examine the relationship between eating habits and prostate cancer (PC) risk. While the associations between PC risk with the glycemic index and Mediterranean score have been reviewed, no meta-analysis is currently available on dietary patterns defined by “a posteriori” methods. A literature search was carried out (PubMed, Web of Science) to identify studies reporting the relationship between dietary patterns and PC risk. Relevant dietary patterns were selected and the risks estimated were calculated by a random-effect model. Multivariable-adjusted odds ratios (ORs), for a first-percentile increase in dietary pattern score, were combined by a dose-response meta-analysis. Twelve observational studies were included in the meta-analysis which identified a “Healthy pattern” and a “Western pattern”. The Healthy pattern was not related to PC risk (OR = 0.96; 95% confidence interval (CI): 0.88–1.04) while the Western pattern significantly increased it (OR = 1.34; 95% CI: 1.08–1.65). In addition, the “Carbohydrate pattern”, which was analyzed in four articles, was positively associated with a higher PC risk (OR = 1.64; 95% CI: 1.35–2.00). A significant linear trend between the Western (*p* = 0.011) pattern, the Carbohydrate (*p* = 0.005) pattern, and the increment of PC risk was observed. The small number of studies included in the meta-analysis suggests that further investigation is necessary to support these findings.

## 1. Introduction

Prostate cancer (PC) is the second most common cancer in men after lung cancer, with more than 1.1 million new cases and over 307,000 deaths estimated worldwide in 2012 [1]. Almost 70% of PC cases occur in more developed regions and its incidence varies more than 25-fold in different geographical areas. Incidence rates are higher in Northern and Western Europe compared to Central and Eastern countries. In Northern America, PC incidence is about 10 times higher than in Asia [1]. Although these regional differences could be due to both race (genetic factors) and screening programs for early diagnosis, evidence suggests that environmental and dietary factors may also play important roles in prostate carcinogenesis [2,3].

Several epidemiological studies have explored the association of dietary habits on PC risk, and meta-analysis has recently summarized the association of individual foods and nutrients with PC risk. Indeed, significant preventive effects have been found for the intake of allium vegetables [4], carrots and coffee [5,6]; whereas, inconsistent correlation has been observed between PC risk and the consumption of tomatoes and lycopene [7,8], tea [9], fruits and vegetables [10], fiber [11], fat [12], red meat, processed meat, and seafood [13]. On the other hand, dairy products, calcium, and eggs seem to act as risk factors for PC [13,14]. In any case, as recently reported by the World Cancer Research Fund International/American Institute for Cancer Research, the role of individual foods and nutrients on PC risk is still limited and controversial [15].

Rather than studying the individual foods and nutrients, recently, dietary patterns have been applied in nutritional epidemiology to examine the relationship between diet and chronic diseases [16,17]. This strategy allows the study of the effects of overall dietary habits in a way more closely related to the real conditions in which foods and nutrients are consumed. Several statistical methods have been used to define dietary patterns. They can be distinguished in “a posteriori” methods, such as factor analysis (FA), cluster analysis (CA), principal component analysis (PCA), and principal component factor analysis (PCFA), which generate patterns (i.e., Western, prudent, healthy patterns) on the basis of available dietary data obtained directly from the studied population; and in “a priori” approaches which derive dietary indices and/or scores (i.e., Glycemic index, Mediterranean score) on the basis of previous knowledge of the healthy or unhealthy effects of various diet constituents [18]. In the last few years, several epidemiological studies have used these methods to estimate the relationships between different dietary patterns and PC risk. However, while the association between both the “Glycemic index” and “Mediterranean score” have been recently reviewed and estimated; to the best of our knowledge, no meta-analysis is currently available considering the effect of dietary patterns defined by “a posteriori” methods on PC risk [19,20].

In this systematic review and meta-analysis, we selected studies addressing the correlation between the different dietary patterns defined using “a posteriori” methods and PC risk, and we used them to provide a quantitative estimation of the association.

## 2. Materials and Methods

### 2.1. Literature Search Strategy and Selection Criteria

We carried out a comprehensive literature search, without restrictions, up to December 2015 through PubMed (http://www.ncbi.nlm.nih.gov/pubmed/) and Web of Science (http://wokinfo.com/) databases. The original articles on the association between dietary patterns and PC risk were identified using the following search key words: (“neoplasm” OR “cancer” OR “neoplastic disease”) AND (“prostate” OR “prostatic”) AND (“dietary pattern” OR “eating pattern” OR “food pattern” OR “dietary habit” OR “diet” OR “dietary”) AND (“factor analysis,” OR “principal component analysis” OR “cluster analysis” OR “clustering” OR “reduced rank regression” OR “diet diversity” OR “diet variety” OR “quality” OR “index” OR “indices” OR “scores”). Furthermore, the reference lists of included articles and recent significant reviews were manually examined to identify additional relevant publications. The standard procedure for conducting and reporting meta-analysis, according to the guidelines from the Meta-analysis Of Observational Studies in Epidemiology (MOOSE) group, were followed [21]. Reviews and pooled analyses were excluded, although, if they have been useful for background information. Potential identified articles were included if they met the following criteria: (i) used a case-control or prospective study design; (ii) evaluated the association between dietary patterns derived by “a posteriori” methods and PC risk; (iii) presented odds ratio (OR), relative risk (RR), or hazard ratio (HR) estimates with 95% confidence intervals (CIs). In the presence of several publications from the same study, the publication with the largest number of cases was selected. For each potential included study, two investigators independently carried out the selection evaluation, data abstraction, and quality assessment. Disagreements were resolved by discussion or in consultation with a third author.

### 2.2. Data Abstraction and Quality Assessment

From the selected studies, we extracted the following information: study design, first author’s last name, year of publication, geographical area and country, sample size (when possible, number of cases and controls; cohort size and incident cases), age, duration of follow-up for cohort studies, dietary assessment and dietary pattern identification methods (FA, CA, PCA and PCFA), characteristics of the dietary assessment method, name of the dietary pattern type and its characteristics, cutoff points of the different categories of adherence to the dietary pattern (dichotomy, tertile, quartile and quintile), risk estimates with 95% confidence intervals for the different categories of adherence, and *p*-value for trend and confounding factors adjustment. When multiple estimates were reported in the article, we pulled out those that adjusted for the most confounding factors. The study quality was assessed by a 9-star system, based on the Newcastle–Ottawa Scale method [22]. Therefore, the full score was 9 and a total score of ≥7 was used to indicate a high-quality study. To avoid selection bias, no study was excluded because of these quality criteria.

### 2.3. Statistical Analysis

The overall effect-size statistic estimated was the average of the logarithm of the observed odds ratio (approximated to RR, when necessary) associated with the highest versus the lowest level of consumption. We used the random effects model to calculate the summary OR and 95% confidence intervals. A two-tailed *p* < 0.05 was considered statistically significant. We restricted the analysis to the “a posteriori” dietary patterns. In addition, we carried out a dose-response meta-analysis to compute the trend across categories. The linear increase in PC risk, per percentile increase in dietary pattern, was estimated using the method proposed by Greenland and Longnecker [23], which accounts for the correlation between risk estimates for separate exposure levels depending on the same reference group, when possible. For studies with non-zero or different exposure dose as reference, we adjusted the values following Liu et al. [24]. We estimated the distribution of cases or controls—or person’s years in studies—that did not report these, but we reported the total number of cases or controls—or person’s years in studies—if the results were analyzed by quantiles (dividing the total number of person’s years in studies by the number of reported quantiles). The study-specific trends were then combined according to the principles of multivariate random-effects meta-analysis. The two most common dietary patterns, which had similar factor loading of principle components, were identified in 9 case-control studies [25,26,27,28,29,30,31,32,33] and 3 cohort studies [34,35,36]. The first pattern, named “Healthy pattern” was characterized by a high loading of vegetables and fruits, poultry, fish, and whole grains. The selected articles were labeled as “Healthy” [25,28,32], “Vegetable” [26,30,34,36], “Prudent”, and “Vitamins and Fiber” [27,29,31,33,35]. The second pattern, named “Western pattern”, had a high loading of red meat, processed meat, eggs, and sweets. The included articles labeled it as “Traditional Western/Processed diet” [25], “Western” [26,27,29,32,35], “Organ meat and fast food” [28], “Meat” [30], “Animal Products” [31], “Traditional” [33], “Red meat-starch”, and “Meat and Potatoes” [34,36]. In addition, a “Carbohydrate pattern”, characterized by a high loading of bread, pasta, and rice, was identified in four articles which labeled it as “Carbohydrate” [28,33], “Refined carbohydrate” and “Starch-rich” [30,31]. The chi-square-based Cochran’s *Q* statistic and the *I*^2^ statistic were used to evaluate heterogeneity in results across studies [37]. For the *Q* statistic, a *p*-value of < 0.1 was considered to be representative of statistically significant heterogeneity. The *I*^2^ statistic yields results ranged from 0% to 100% (*I*^2^ = 0%–25%, no heterogeneity; *I*^2^ = 25%–50%, moderate heterogeneity; *I*^2^ = 50%–75%, large heterogeneity; and *I*^2^ = 75%–100%, extreme heterogeneity) [38]. Results of the meta-analysis may be biased if the probability of a study being published is dependent on its results. We used the methods of Begg and Mazumdar, and Egger et al. to detect publication bias [39,40]. Both methods tested for funnel plot asymmetry, the former being based on the rank correlation between the effect estimates and their sampling variances, and the latter on a linear regression of a standard normal deviate on its precision. If a potential bias was detected, we further conducted a sensitivity analysis to assess the robustness of combined effect estimates, and the possible influence of the bias, and to have the bias corrected. We also conducted a sensitivity analysis to investigate the influence of a single study on the overall risk estimate, by omitting one study in each turn. We considered the funnel plot to be asymmetrical, if the intercept of Egger’s regression line deviated from zero, with a *p*-value of <0.05. Subgroup analyses were conducted for the case-control and cohort studies. The ProMeta Version 2.0 statistical program (Internovi, Via Cervese, 47522, Cesena, Italy) and packages dosresmeta 1.3.2. for R 3.1.2. (R Foundation for Statistical Computing, Vienna, Austria) were used for the analyses [41]. All reported *p* values are from two-sided statistical tests, and differences with *p* ≤ 0.05 were considered significant.

## 3. Results

### 3.1. Study Selection

**Figure 1:**
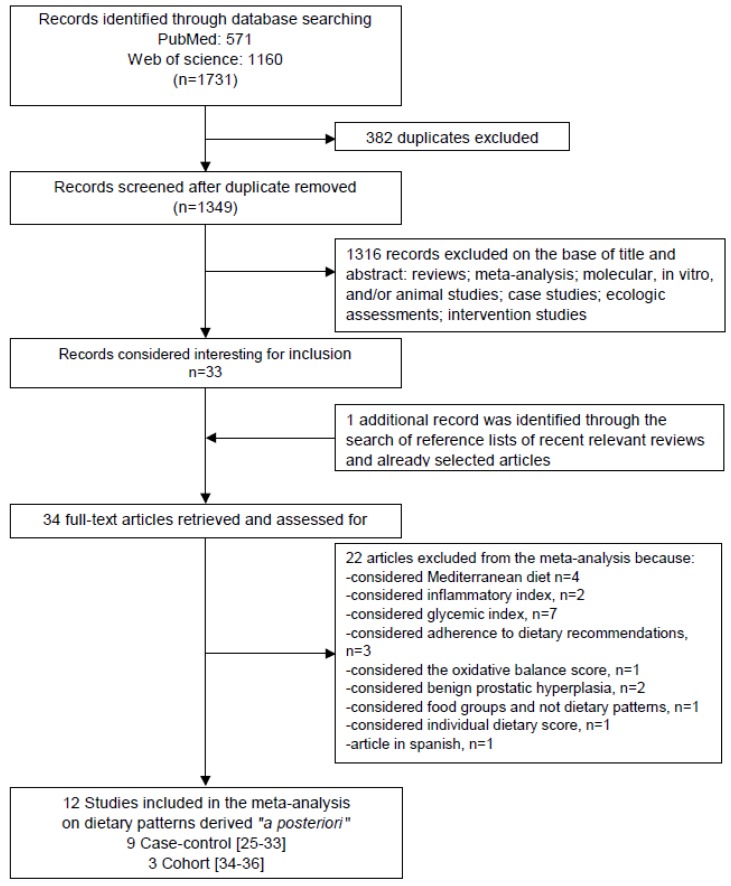
Flow diagram of systematic literature search on dietary patterns and prostate cancer risk.

From the primary literature research through PubMed (*n* = 571) and Web of Science (*n* = 1160) databases, and after removing duplicates (*n* = 382), we identified 1349 records for title and abstract revision (Figure 1). Among the 1349 articles screened, 1316 were excluded because they were not observational epidemiological studies. Thirty-three articles were subjected to full-text revision. Hand searching of reference lists of both selected articles and recent relevant reviews led to the identification of one additional item. Subsequently, 22 papers were excluded because they did not meet the inclusion criteria as follows: Four studies were on adherence to the Mediterranean diet, two were considered the inflammatory index, seven were considered the glycemic index, three studies were on adherence to dietary recommendations, one was considered the oxidative balance score, two were considered benign prostatic hyperplasia, one was considered food groups and not dietary patterns, one was considered individual dietary score, and two articles showed the results of the same study (so we did not consider the one in Spanish). Therefore, at the end of the selection process, 12 studies met the inclusion criteria (Figure 1) and were enclosed for the identification of the different dietary patterns in the systematic review and meta-analysis [25,26,27,28,29,30,31,32,33,34,35,36].

### 3.2. Study Characteristics and Quality Assessment

Of all the selected papers, nine were case-control studies and three were cohort studies. General characteristics of case-control and cohort studies are shown in Table 1 and Table 2, respectively.

Case-control studies were published between 2005 and 2015. Two of them were population-based [26,33] and seven were hospital-based [25,27,28,29,30,31,32]. Two studies were conducted in Uruguay [27,29] and Jamaica [28,30], and one each in Canada [25], Australia [26], Italy [31], Iran [32] and Argentina [33]. Cohort studies were published between 2004 and 2009; two were conducted in the United States [34,35] and one was conducted in Australia [36]. All of the 12 included articles used a food frequency questionnaire (FFQ) to collect dietary information (64 to 131 items). Two studies reported the association of PC risk with two different dietary patterns [32,35], two studies considered three dietary patterns [26,34], six studies considered four dietary patterns [25,27,28,30,33,36] and two studies considered five different dietary patterns [29,31]. All of the 12 selected articles identified both a “Healthy pattern” and a “Western pattern”, while four studies identified a “Carbohydrate pattern”, too. On the other hand, only two studies identified a “Drinker pattern”, and they are small in number to conduct a meta-analysis on them [27,29]. It is a well-known fact that BMI is related to PC risk. Nevertheless, three studies [25,32,34] did not include BMI as an adjusted variable for the risk analysis. However, in a preliminary analysis, all of them have considered the BMI as a potential confounder, but they have not found a significant association with prostate cancer risk. Study-specific quality scores are summarized in supplementary materials Tables S1 and S2 for case-control and cohort studies, respectively (available online). For case-control studies, the range of quality score was from 5 to 8 (median: 8, mean ± SD: 7.1 ± 1.2) and high-quality was reached by six studies [26,27,29,30,31,33], while all three cohort studies reached a quality score of 8 [34,35,36].

### 3.3. Meta-Analysis

**Figure 2:**
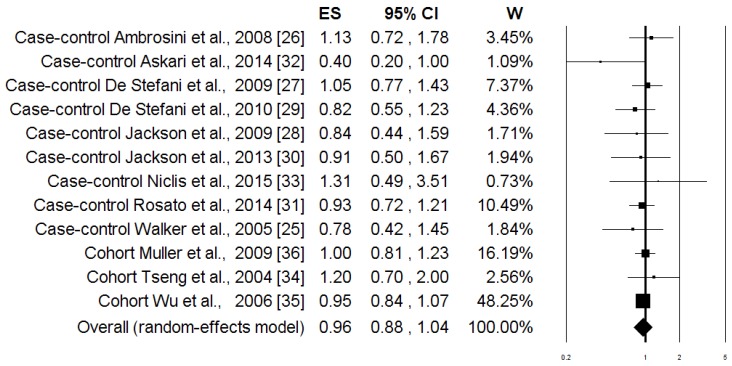
Forest plot of the highest compared with the lowest categories of intake of the “Healthy” dietary pattern and prostate cancer risk.

**Figure 3:**
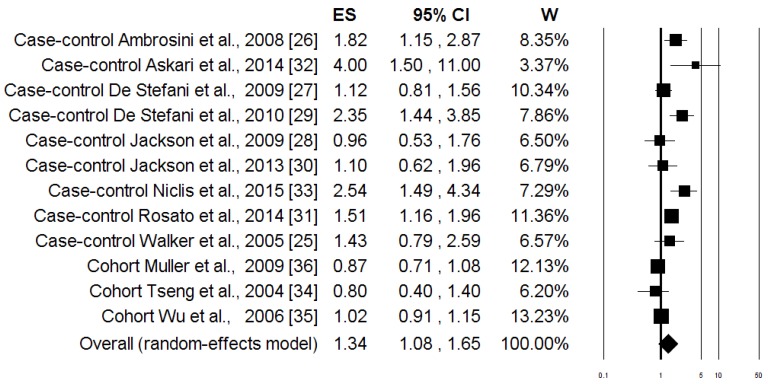
Forest plot of the highest compared with the lowest categories of intake of the “Western” dietary pattern and prostate cancer risk.

**Figure 4:**
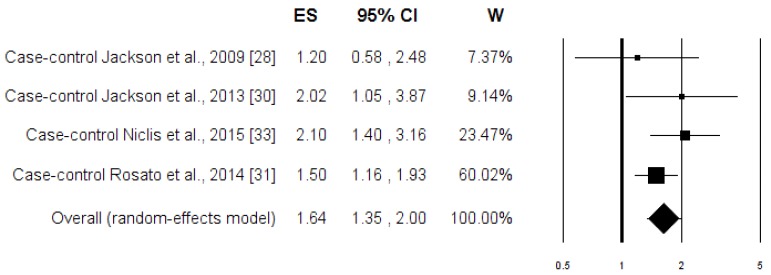
Forest plot of the highest compared with the lowest categories of intake of the “Carbohydrate” dietary pattern and prostate cancer risk.

The associations between the highest intake compared to the lowest intake categories of the “Healthy pattern” and PC risk are shown in Figure 2. When data from all of the studies were pooled together, there was no evidence of a significant reduction in PC risk associated with the Healthy pattern (OR = 0.96; 95% CI: 0.88, 1.04; *p* = 0.284). Similar results were obtained when the analysis was carried out separately for case-control and cohort studies (Table 3). The heterogeneity was not apparent in any case (Table 3). Figure 3 shows the associations between the highest, compared to the lowest, intake categories of the “Western pattern” and PC risk for the all studies included in the meta-analysis. There was a statistically significant increment of PC risk associated with the Western pattern (OR = 1.34; 95% CI: 1.08, 1.65; *p* = 0.007), but the heterogeneity was rather high (*I*^2^ = 74.63%, *p* = 0.0001) (Table 3). The Subgroup analysis, according to the study design, showed that heterogeneity remains evident in the case-control studies (*I*^2^ = 54.61%, *p* = 0.024), where an increase in the risk of PC was shown (OR = 1.58; 95% CI: 1.25; 2.01; *p* = 0.0001). Otherwise, there was no evidence of heterogeneity (*I*^2^ = 4.14%, *p* = 0.352), and there was no evidence of a difference in the risk of PC (OR = 0.97; 95% CI: 0.87, 1.08; *p* = 0.352) (Table 3) in the cohort studies. In Figure 4, the associations between the highest and lowest intake categories of “Carbohydrate pattern” and PC risk for the four case-control studies included in the meta-analysis are reported. It was observed that a significant increment of PC risk was associated with the Carbohydrate pattern (OR = 1.64; 95% CI: 1.35, 2.00; *p* = 0.0001), in the absence of heterogeneity (*I*^2^ = 0.00%, *p* = 0.393) (Table 3). 

### 3.4. Dose-Response Analysis

**Figure 5:**
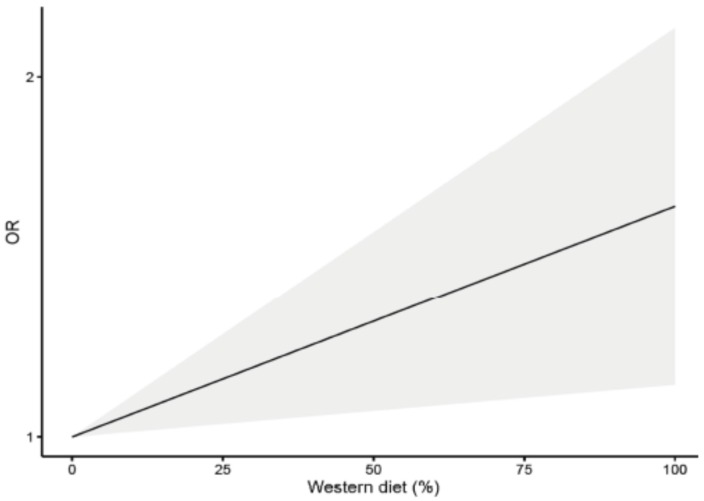
Dose-response plot of the linear relation between the intake of the “Western” dietary pattern and prostate cancer risk.

We started analyzing papers which reported complete observations. Eight studies were considered, six case control studies [25,26,29,30,31,33] and two cohort studies [34,35]. Figures S1 and S2 (on line) summarize data with estimated trends in OR according to the level of dietary consumption in each study for the Healthy and Western pattern, respectively. For the “Healthy diet”, a clear trend was not evident. In fact, the result didn’t show an increase in PC risk. The summary OR for each percentile increment was 0.999 (95% CI: 0.998, 1.001) with no evidence of heterogeneity (*I*^2^ = 0%, *p* = 0.82). Furthermore, the linear dose-response curves showed a slightly inverse, but not significant (*p* = 0.345), association between Healthy diet consumption and PC risk. In contrast, for the Western diet, PC risk increased with the percentile of dietary adherence. The summary OR was 1.004 (95% CI: 1.001, 1.008) for each percentile increment in the intake of Western diet, with high heterogeneity (*I*^2^ = 74.9%, *p* = 0.001). A linear trend was evident (*p* = 0.011) as shown in Figure 5. The model predicted values for 20% percentiles which corresponded to 1.093 (95% CI: 1.021, 1.170). Using the missing imputation previously explained, we repeated the analysis with the inclusion of two studies [28,29,30,31,32,33,34,35,36]. No significant changes occurred for the Healthy pattern. The summary OR for the Western diet was 1.003 (95% CI: 1.001, 1.006; *p* = 0.021) with always high evidence of heterogeneity (*I*^2^ = 73.7%, *p* = 0.001). Further, considering studies without missing data, we conducted a subgroup analysis for several case-control studies. We did not repeat the analysis for the selected cohort studies because only two of them were available for the dose-response analysis. A linear trend for the Western pattern was still evident in the case-control studies (*p* = 0.002). The summary OR was 1.007 (95% CI: 1.003, 1.010). The heterogeneity was still quite high (*I*^2^ = 51.1%, *p* = 0.069), but less than in the dose-response analysis—which considered the whole sample. In a sensitivity analysis, excluding one study at a time, no particular differences in the results arose. Even if only three studies identified a “Carbohydrate pattern” and showed complete data [30,31,33], we also conducted a dose-response meta-analysis on this dietary set. The data detected a linear trend statistically significant (*p* = 0.005). The estimated OR was 1.007 (95% CI: 1.002, 1.010). There was quite a low heterogeneity (*I*^2^ = 28.7%, *p* = 0.246).

### 3.5. Publication Bias

Funnel plots showed a little evidence of asymmetry and, therefore, of publication bias (Figure S3, online). The corresponding statistical evaluation, by the Begg and Mazumdar’s rank correlation test, demonstrated no significant publication bias in any case (Table 3). On the other hand, the Egger’s linear regression test showed some publication bias for the Healthy pattern in cohort studies (*p* = 0.003) and for the Western pattern in pooled analysis (*p* = 0.045) (Table 3).

### 3.6. Sensitivity Analysis

Sensitivity analyses, investigating the influence of a single study on the PC risk estimate, suggested that results were not substantially modified by removal any single study. In particular, no evident changes were found in the risk estimates after removal of the outlier study of Askari et al. [32] on the Healthy pattern (OR = 0.96; 95% CI: 0.89, 1.05; *p* = 0.399). In addition, the PC risk estimates associated with the Western pattern ranged from 1.26 (95% CI: 1.03–1.54, *p* = 0.013), omitting the study of Niclis et al. [33], to 1.42 (95% CI: 1.13–1.79, *p* = 0.003), omitting the study of Muller et al. [36]. Of note, omitting the study of Niclis et al. [33], in the Western pattern, resulted in the absence of publication bias, as evidenced by both Egger’s regression (*p* = 0.089) and Begg’s rank correlation (*p* = 0.586) tests.

## 4. Discussion

To the best of our knowledge, this is the first systematic review and meta-analysis which considers the effect of different dietary patterns identified by “a posteriori” methods on PC risk. From the selected articles, we identified two very common dietary patterns—“Western” and “Healthy” patterns. These two dietary patterns were present in all of the selected studies. Additionally, four studies reported a further “Carbohydrate” pattern. The results indicated that both of the Western and Carbohydrate dietary patterns were significantly associated to an increase of PC risk, while our analyses showed no association between the Healthy dietary pattern and PC risk.

Several systematic reviews and meta-analyses have recently reported the association between dietary patterns and the risk of cancer in different sites; such as breast [42], colon and rectum [43,44], stomach [45], esophagus, and lung [46,47]. Similar to our results—in some of them, it was found that the consumption of a Western dietary pattern was positively associated to an increment of cancer risk in the colon [43] colorectal, and stomach [44,45]. On the other hand, no significant correlation was evidenced for breast [42], rectal, and esophagus cancers [43,46]. The identified Western pattern is characterized by a high consumption of red meat, processed meat, eggs, and sweets. These foods may be plausibly responsible, among the others, for the pro-carcinogenic properties of this diet. The consumption of processed meat and red meat have been recently classified by the International Agency for Research on Cancer (IARC) as “carcinogenic to humans” (Group 1) and “probably carcinogenic to humans” (Group 2A), respectively [48]. The carcinogenic activity of meat may be mediated by the presence of mutagenic compounds; such as heterocyclic amines and polycyclic aromatic hydrocarbons, which are formed during cooking at high temperatures or over flame. However, a recent meta-analysis failed to show any positive correlation between red and processed meat, cooking methods, and the concentration of heterocyclic amines with the risk of PC [49]. This observation suggests that the Western dietary pattern may have more complex interactions with PC risk than those expected for the red meat and processed meat considered as individual foods.

Regarding the Healthy pattern, previous meta-analyses showed an evident and significant inverse correlation with cancer risk in all anatomic sites considered, with the exclusion of the rectum [42,43,44,45,46,47]. In contrast, our data suggested a small reduction of PC risk (4%, when comparing the highest with the lowest intake categories) which was not statistically significant. These results may be difficult to explain; since the Healthy pattern is characterized by a high load of vegetables and fruits, which are a rich source of antioxidants with potential chemo-preventive activities. However, it should be considered that, in this dietary pattern, other foods such as poultry, fish and whole grains were also included. In any case, our results agree with previous meta-analyses to show no effect of single-vegetable foods and fish on PC risk [7,8,9,10,11,12,13].

In our study, we also found a Carbohydrate dietary pattern which was associated with a statistically significant (64%) increment of PC risk. This result is of particular interest, considering that the Carbohydrate pattern had high-factor loading for bread, pasta, and rice. However, this conclusion should be interpreted with caution, since it was obtained from only four studies. Further, epidemiological evidences are necessary to confirm this trend considering that a recent meta-analysis also showed no correlation between consumption of dietary fiber, whole grains, and carbohydrates with PC risk [50].

One of the great limitations of meta-analysis is that the results are combined from studies conducted with different methods in different populations, resulting in heterogeneity. In our analysis, heterogeneity was more evident in the results regarding the “Western pattern”. This could be due to the difficulty in characterizing this pattern. Moreover, a possible misclassification within the considered dietary patterns may be present. Factor analysis and/or principal component analysis are subjective techniques with opportunities for variation at almost every step [16]. Other limitations of our meta-analysis could be linked to the fact that pooled findings were directly driven by the included studies, which have their weaknesses relative to study design. In addition, risk estimates were adjusted for different potential confounders.

## 5. Conclusions

In conclusion, we pooled information from twelve studies which identified different “a posteriori” dietary patterns in terms of single food or nutrient items mainly correlated to them. We selected two main dietary patterns, which were analyzed in all of the studies, and a dietary pattern was reported in just four of them. From the dietary pattern named as “Healthy”, mainly based on a high consumption of vegetables and fruits, poultry, fish, and whole grains, a statistical significance association with PC risk was not highlighted. Different results emerged from the dietary patterns named as “Western” and “Carbohydrate”, characterized by a high loading of red meat, processed meat, eggs, sweets and bread, pasta and rice, respectively. An increase in PC risk was pointed out in the highest, compared to the lowest, categories of dietary patterns in all pooled studies and in the dose-response meta-analysis, even if the heterogeneity was quite high. The high heterogeneity may be correlated to the wide variability between studies regarding different aspects of dietary data collection and analysis; such as food items and food sub-types considered, the different dietary patter identified, and the various and not uniformly adjusted confounding factors used to calculate the risk. As a consequence of this, and considering also the small number of cohort studies so far published, further investigations are necessary to support these findings.

## Figures and Tables

**Table 1:** Main characteristics of the case-control studies included in the systematic review and meta-analysis of dietary patterns and prostate cancer risk.

Author, Year Location	Case/Control Age Period	Dietary Pattern Assessment and Identification Method	Dietary Pattern Type and Characteristics	Pattern Score	OR (95% CI)	*p* for Trend	Confounding Factor Adjusted
Walker, 2005 [25] Canada	80/334 HB ^1^ 50–80 years (mean: 65.0 years cases; 63.6 years controls) (1997–1999)	67-item FFQ ^2^ (2 years before), SA ^3^ 34 food groups PCA ^4^ (in controls) Varimax rotation, EIG ^5^ > 1.5 Loading > 0.25 4 factors, VE ^6^ 10.51%	***1. Healthy living*:** vegetables, fruits, whole grains, fish, and poultry	Tertile 1	1.00 (Ref.)	0.45	Age, physical activity as a teen, current smoking and alcohol intake
				Tertile 2	0.99 (0.55–1.78)		
				Tertile 3	0.78 (0.42–1.45)		
			***2. Traditional Western*:** red meats, processed meats, milk, sweets, and hard liquor	Tertile 1	1.00 (Ref.)	0.22	
				Tertile 2	1.00 (0.53–1.88)		
				Tertile 3	1.43 (0.79–2.59)		
			***3. Processed diet*:** processed meats, red meats, organ meats, refined grains, onions and tomatoes, vegetable oils and juices, bottled water, and soft drinks	Tertile 1	1.00 (Ref.)	0.003	
				Tertile 2	2.11 (1.06–4.22)		
				Tertile 3	2.75 (1.40–5.39)		
			***4. Beverages*:** tap water, “other” beverages including soft drinks and fruit juices, potatoes, poultry and margarine/inversely associated with beer, liquor, wine, and cream for coffee	Tertile 1	1.00 (Ref.)	0.54	
				Tertile 2	0.68 (0.37–1.25)		
				Tertile 3	0.84 (0.47–1.51)		
Ambrosini, 2008 [26] Australia	546/447 PB ^7^ 40–75 years	101-item FFQ (10 years before), SA PCA (in controls) Varimax rotation, EIG > 1, scree plots Loading > 0.3 3 factors, VE 29.2%	***1. Vegetable*:** all vegetables listed in the FFQ (including fresh and tinned tomatoes) plus jam, honey, and apples	Quartile 1	1.00 (Ref.)	0.46	Age, BMI ^8^, energy intake, and paternal history of prostate cancer
				Quartile 2	1.03 (0.71–1.50)		
				Quartile 3	1.26 (0.85–1.89)		
				Quartile 4	1.13 (0.72–1.78)		
			***2. Western*:** full cream milk, white bread, cakes, potato crisps, French fries (chips), eggs, red and processed meats, hamburgers, fried or takeaway fish, and full alcohol beer	Quartile 1	1.00 (Ref.)	0.02	
				Quartile 2	1.42 (0.98–2.06)		
				Quartile 3	1.32 (0.89–1.97)		
				Quartile 4	1.82 (1.15–2.87)		
			***3. Health-conscious*:** steamed and grilled fish, tinned fish, chicken, rice, pasta, legumes, and tofu; bean sprouts, nuts, yogurt, ricotta cheese, red wine, and white wine	Quartile 1	1.00 (Ref.)	0.97	
				Quartile 2	1.24 (0.86–1.80)		
				Quartile 3	1.02 (0.70–1.48)		
				Quartile 4	1.06 (0.72–1.58)		
De Stefani, 2009 [27] Uruguay	345/2532 HB (1996–2004)	64-item FFQ, IA ^9^ 17 food groups PCA (in controls) Varimax rotation, Loading > 0.39 4 factors, VE 36.6%	***1. Prudent*:** poultry, fish, fresh vegetables, cooked vegetables, and total fruits	Tertile 1	1.00 (Ref.)	0.76	Age, residence, urban/rural status, education, BMI, smoking status, years since stopping, number of cigarettes/dayamong current smokers, total energy intake and all the dietary patterns
				Tertile 2	1.01 (0.75–1.37)		
				Tertile 3	1.05 (0.77–1.43)		
			***2. Traditional*:** total grains, all tubers, desserts, and dairy foods	Tertile 1	1.00 (Ref.)	0.37	
				Tertile 2	1.12 (0.80–1.55)		
				Tertile 3	1.20 (0.81–1.78)		
			***3. Western*:** fried red meat, barbecue meat, processed meat, and eggs	Tertile 1	1.00 (Ref.)	0.42	
				Tertile 2	1.36 (1.01–1.81)		
				Tertile 3	1.12 (0.81–1.56)		
			***4. Drinker*:** alcoholic beverages such as beer, wine, and hard liquor	Tertile 1	1.00 (Ref.)	0.29	
				Tertile 2	0.69 (0.51–0.92)		
				Tertile 3	0.86 (0.64–1.17)		
Jackson, 2009 [28] Jamaica	204/204 HB (2004–2007)	FFQ, IA 33 food groups PCFA Varimax rotation, EIG > 1 Loading > 0.4 4 factors, VE 24.5%	***1. Healthy*:** vegetables, fruits, peas and beans	Tertile 1	1.00 (Ref.)	Not reported	Age, family history of prostate cancer, education, BMI, smoking, alcohol and total energy intake
				Tertile 2	0.72 (0.39–1.32)		
				Tertile 3	0.84 (0.44–1.59)		
			***2. Carbohydrate*:** white bread and refined cereals, poultry, rice/pasta, starchy roots, and tubers	Tertile 1	1.00 (Ref.)		
				Tertile 2	1.28 (0.70–2.43)		
				Tertile 3	1.20 (0.58–2.48)		
			***3. Sugary foods*:** sweet baked products and non-diet drink	Tertile 1	1.00 (Ref.)		
				Tertile 2	0.88 (0.49–1.62)		
				Tertile 3	0.75 (0.40–1.38)		
			***4. Organ meat and fast food*:** high-fat dessert, organ meat, fast food, and salty snacks	Tertile 1	1.00 (Ref.)		
				Tertile 2	1.31 (0.73–2.33)		
				Tertile 3	0.96 (0.53–1.76)		
De Stefani, 2010 [29] Uruguay	345/690 HB 45–89 years (1996–2004)	64-item FFQ, IA 21 food groups PCA Quartimax orthogonal Scree plot 5 factors, VE 38.4%	***1. Prudent*:** raw vegetables, citrus fruits, other fruits, and tea	Quartile 1	1.00 (Ref.)	0.40	Education, occupation, family history of prostate cancer among first-degree relatives, BMI, tobacco smoking, total energy intake and each pattern for the others
				Quartile 2	0.96 (0.66–1.41)		
				Quartile 3	0.96 (0.66–1.42)		
				Quartile 4	0.82 (0.55–1.23)		
			***2. Traditional*:** lamb, dairy foods, cooked vegetables, and all tubers	Quartile 1	1.00 (Ref.)	0.01	
				Quartile 2	1.62 (1.07–2.45)		
				Quartile 3	1.87 (1.22–2.87)		
				Quartile 4	1.85 (1.16–2.94)		
			***3. Substituter*:** poultry and fish and a negative loading for lamb consumption	Quartile 1	1.00 (Ref.)	0.58	
				Quartile 2	1.38 (0.94–2.02)		
				Quartile 3	1.35 (0.91–2.01)		
				Quartile 4	1.07 (0.70–1.65)		
			***4. Drinker*:** mate, beer, wine, and hard liquor	Quartile 1	1.00 (Ref.)	0.42	
				Quartile 2	0.79 (0.54–1.16)		
				Quartile 3	0.89 (0.61–1.32)		
				Quartile 4	1.18 (0.78–1.78)		
			***5. Western*:** beef, processed meat, boiled eggs, fried eggs, and total grains	Quartile 1	1.00 (Ref.)	<0.0001	
				Quartile 2	1.41 (0.92–2.17)		
				Quartile 3	2.10 (1.35–3.25)		
				Quartile 4	2.35 (1.44–3.85)		
Jackson, 2013 [30] Jamaica	243/275 HB 40–80 years (2005–2007)	FFQ 33 food groups PCA Varimax rotation EIG > 1, scree plot 4 factors, VE 27.8%	***1.Vegetables & legumes (Healthy)*:** Dark green leafy, yellow vegetable, nuts and seeds, other vegetables, peas and beans, ready-to-eat cereals, fruits	Tertile 1	1.00 (Ref.)	0.766	Age, family history of prostate cancer, education, BMI, smoking, physical activity, total energy intake
				Tertile 2	0.87 (0.49–1.55)		
				Tertile 3	0.91 (0.50–1.67)		
			***2. Fast food*:** fast foods, alcoholic beverages, meal replacements, dairy dessert, fruit juice	Tertile 1	1.00 (Ref.)	0.162	
				Tertile 2	1.12 (0.63–1.96)		
				Tertile 3	0.66 (0.34–1.16)		
			***3. Meat*:** processed meat, eggs, poultry, and starchy fruits, roots, and tubers	Tertile 1	1.00 (Ref.)	0.735	
				Tertile 2	0.88 (0.50–1.57)		
				Tertile 3	1.10 (0.62–1.96)		
			***4. Refined Carbohydrate*:** rice and pasta, sugar sweetened beverages, sweet baked foods (refined carbohydrates), and poultry	Tertile 1	1.00 (Ref.)	0.029	
				Tertile 2	1.65 (0.94–2.90)		
				Tertile 3	2.02 (1.05–3.87)		
Rosato, 2014 [31] Italy	1294/1451 HB 46–74 years (median: 66 years cases; 63 years controls) (1991–2002)	78-items FFQ (2 years before), IA 28 selected nutrients/29 food groups PCFA Varimax rotation EIG ≥ 1, scree plot Loading ≥ 0.63 5 factors, VE 78.26%	***1. Animal Products*:** calcium, phosphorus, riboflavin, animal protein, saturated fatty acids, zinc, and cholesterol Milk and dairy products, eggs, red meat, cheese	Quintile 1	1.00 (Ref.)	0.02	Age, study center, education, BMI, tobacco, alcohol drinkingand family history of prostatecancer in first-degree relatives
				Quintile 3	1.35 (1.04–1.76)		
				Quintile 5	1.51 (1.16–1.96)		
			***2. Vitamins and Fiber*:** vitamin C, total fiber, beta-carotene equivalents, total folate, and soluble carbohydrates Fruits, vegetables, legumes, and olive oil	Quintile 1	1.00 (Ref.)	0.23	
				Quintile 3	1.15 (0.90–1.48)		
				Quintile 5	0.93 (0.72–1.21)		
			***3. Starch-rich*:** starch, vegetable protein, and sodium Bread, pasta and rice	Quintile 1	1.00 (Ref.)	<0.001	
				Quintile 3	1.16 (0.89–1.49)		
				Quintile 5	1.50 (1.16–1.93)		
			***4. VUFA ^10^*:** linoleic acid, vitamin E, and linolenic acid Specified and unspecified seed oils, leafy vegetables, and olive oil	Quintile 1	1.00 (Ref.)	0.41	
				Quintile 3	1.21 (0.94–1.55)		
				Quintile 5	1.16 (0.89–1.51)		
			***5. AUFA ^11^*:** polyunsaturated fatty acids and vitamin D Fish and offals	Quintile 1	1.00 (Ref.)	0.02	
				Quintile 3	1.05 (0.81–1.36)		
				Quintile 5	1.32 (1.02–1.70)		
Askari, 2014 [32] Iran	50/100 HB 40–78 years (cases) 43–71 years (controls)	125-item FFQ, IA PCA Varimax rotation Interpretability and scree plot EIG > 1.9 2 factors	***1. Western diet*:** sweets and desserts, organ meat, snacks, tea and coffee, French fries, salt, carbonated drinks, red or processed meat	Two categories (high 2nd median vs. low 1st median)	4.00 (1.50–11.00)		Smoking, diabetes, energy intake
			***2. Healthy diet*:** legumes, fish, dairy products, fruits and fruit juice, vegetables, boiled potatoes, whole cereal, and egg		0.40 (0.20–1.00)		
Niclis, 2015 [33] Argentina	147/300 PB 48–89 years (cases) 46–89 years (controls)	125-item FFQ, IA 24 food groups PCA Varimax rotation Interpretability and scree plot EIG > 1.0 Loading ≥ 0.40 4 factors, VE 31.52%	***1. Traditional*:** fatty red meats, offal, processed meat, starchy vegetables, added sugars and sweets, candies, fats, and vegetable oils	Quartile 1	1.00 (Ref.)	0.048	Age, BMI, energy intake, occupational exposure, family history of cancer
				Quartile 2	1.60 (0.97–2.66)		
				Quartile 3	1.73 (1.17–2.57)		
				Quartile 4	2.54 (1.49–4.34)		
			***2. Prudent*:** nonstarchy vegetables, whole grains, and low loading for alcoholic drinks	Quartile 1	1.00 (Ref.)	0.926	
				Quartile 2	0.70 (0.39–1.26)		
				Quartile 3	0.84 (0.54–1.31)		
				Quartile 4	1.31 (0.49–3.51)		
			***3. Carbohydrate*:** sodas/juices and bakery products	Quartile 1	1.00 (Ref.)	0.069	
				Quartile 2	1.76 (1.25–2.48)		
				Quartile 3	2.67 (0.98–7.35)		
				Quartile 4	2.10 (1.40–3.16)		
			***4. Cheese*:** cheese and low loading for fish	Quartile 1	1.00 (Ref.)	0.720	
				Quartile 2	1.48 (0.69–3.20)		
				Quartile 3	1.34 (0.84–2.16)		
				Quartile 4	1.02 (0.54–1.93)		

^1^ Hospital Based; ^2^ Food Frequency Questionnaire; ^3^ Self-administered; ^4^ Principal Component Analysis; ^5^ Eigenvalues; ^6^ Variance Explained; ^7^ Population Based; ^8^ Body Mass Index; ^9^ Interviewer Administered; ^10^ Vegetable Unsaturated Fatty Acids; ^11^ Animal Unsaturated Fatty Acids.

**Table 2:** Main characteristics of the cohort studies included in the systematic review and meta-analysis of dietary patterns and prostate cancer risk.

Author, Year Location	Subjects Cohort Age Incident Cases Follow-up (Period)	Dietary Pattern Assessment and Identification Method	Dietary Pattern Type and Characteristics	Pattern Score	RR (95% CI)	*p* for Trend	Confounding Factor Adjusted
Tseng, 2004 [34] USA	3779 25–74 years (mean 58 year) 136 cases mean follow-up 7.6 years (1982–1992)	105-item FFQ ^1^ PCA ^2^ (Varimax rotation, interpretability, EIG ^3^ > 1.0, Scree plots) Loading > 0.2 3 factors, VE ^4^ 10.8%	***1. Vegetable-fruit*:** vegetables, fruits, fish, and shellfish	Tertile 1	1.00 (Ref.)	0.64	Age, race, poverty census enumeration district, family income, region, residence, education, sun exposure, physical activity, smoking, alcohol, energy intake
				Tertile 2	1.50 (0.9–2.3)		
				Tertile 3	1.20 (0.7–2.0)		
			***2. Red meat-starch*:** red meats, potatoes, salty snacks, cheese, sweets, and desserts	Tertile 1	1.00 (Ref.)	0.37	
				Tertile 2	0.70 (0.5–1.2)		
				Tertile 3	0.80 (0.4–1.4)		
			***3. Southern*:** beans, rice, and such traditionally Southern United States foods as cornbread, grits, sweet potatoes, and okra	Tertile 1	1.00 (Ref.)	0.08	
				Tertile 2	0.90 (0.6–1.4)		
				Tertile 3	0.60 (0.4–1.1)		
Wu, 2006 [35] USA	47,725 40–75 years 3002 cases follow-up 15 years (1986–2000)	131-item FFQ 40 food groups, FA ^5^ (Varimax rotation, interpretability, EIG > 1.0, Scree test) Loading > 0.3 2 factors, VE 17.4%	***1. Prudent*:** fruits, vegetables, legumes, whole grains, fish, and poultry	Quintile 1	1.00 (Ref.)	0.37	Age, height, smoking, family history of prostate cancer, race, history of vasectomy, vigorous exercise, BMI ^6^, alcohol intake, total energy intake
				Quintile 3	1.10 (0.98–1.24)		
				Quintile 5	0.95(0.84–1.07)		
			***2. Western*:** red meat, processed meat, butter, eggs, refined grains and high-fat dairy	Quintile 1	1.00 (Ref.)	0.62	
				Quintile 3	1.03 (0.92–1.16)		
				Quintile 5	1.02 (0.91–1.15)		
Muller, 2009 [36] Australia	14,627 34–75 years 1018 cases Mean follow-up 13.6 years (1990–2007)	121-item FFQ FA (Varimax rotation, interpretability, EIG > 2) Loading > 0.3 4 factor, VE 67%	***1. Mediterranean*:** some meats, vegetables, and fruits, and avoidance of cakes and sweet biscuits	Quartile 1	1.00 (Ref.)	0.9	Age, total energy intake and ethnicity. Further adjustment for BMI, physical activity, smoking, alcohol intake, and education did not change estimated HRs or 95% CIs materially.
				Quartile 2	0.93 (0.79–1.11)		
				Quartile 3	1.14 (0.95–1.37)		
				Quartile 4	0.93 (0.74–1.18)		
			***2. Vegetable*:** high intake of vegetables	Quartile 1	1.00 (Ref.)	0.5	
				Quartile 2	1.11 (0.90–1.36)		
				Quartile 3	1.02 (0.83–1.27)		
				Quartile 4	1.12 (0.90–1.40)		
			***3. Meat & Potatoes*:** high intake of meats and potato cooked in fat	Quartile 1	1.00 (Ref.)	0.2	
				Quartile 2	1.00 (0.84–1.20)		
				Quartile 3	1.04 (0.87–1.24)		
				Quartile 4	0.87 (0.71–1.08)		
			***4. Fruit & Salad*:** high intake of salad greens and fruit	Quartile 1	1.00 (Ref.)	0.6	
				Quartile 2	1.14 (0.96–1.36)		
				Quartile 3	1.10 (0.92–1.32)		
				Quartile 4	1.00 (0.81–1.23)		

^1^ Food Frequency Questionnaire; ^2^ Principal Component Analysis; ^3^ Eigenvalues; ^4^ Variance Explained; ^5^ Factor Analysis; ^6^ Body Mass Index.

**Table 3:** Results of stratified analysis of the risk estimates for the highest compared with the lowest intake categories of different dietary patterns on the basis of study type ^1,2^.

	Combined Risk Estimate		Test of Heterogeneity			Publication Bias	
	Value (95% CI)	*p*	*Q*	*I*^2^ %	*p*	*p* (Egger Test)	*p* (Begg Test)
**Healthy pattern**							
Case-control (*n* = 9) ^3^	0.92 (0.80–1.07)	0.294	6.77	0.00	0.562	0.343	0.404
Cohort (*n* = 3)	0.97 (0.88–1.08)	0.567	0.83	0.00	0.661	0.003	0.117
Pooled ^4^ (*n* = 12)	0.96 (0.88–1.04)	0.284	7.88	0.00	0.724	0.538	0.583
**Western pattern**							
Case-control (*n* = 9)	1.58 (1.25–2.01)	0.0001	17.62	54.61	0.024	0.349	0.677
Cohort (*n* = 3)	0.97 (0.87–1.08)	0.623	2.09	4.14	0.352	0.414	0.602
Pooled ^4^ (*n* = 12)	1.34 (1.08–1.65)	0.007	43.36	74.63	0.0001	0.045	0.583
**Carbohydrate pattern**							
Case-control (*n* = 4)	1.64 (1.35–2.00)	0.0001	2.99	0.00	0.393	0.799	1.000

^1^ The analysis was performed when a number of data ≥3 were available; ^2^ the risk estimates were calculated using the random-effect model; ^3^ in brackets are the number of articles included in the analysis; ^4^ analysis was performed on case-control and cohort studies combined together.

## Supplementary Materials

The following are available online at http://www.mdpi.com/2072-6643/8/10/626/s1, Figure S1: Dose-response plots of the relation between the intake of the “Healthy” dietary pattern and prostate cancer risk in the different studies included in the meta-analysis; Figure S2: Dose-response plots of the relation between the intake of the “Western” dietary pattern and prostate cancer risk in the different studies included in the meta-analysis; Figure S3: Funnel plots of studies included in the meta-analysis evaluating the association between different dietary patterns (Healthy, Western and Carbohydrate) and prostate cancer risk; Table S1: Methodological quality of case-control studies included in the meta-analysis; Table S2: Methodological quality of cohort studies included in the meta-analysis.
