# Supplementary material for: A Western Dietary Pattern Increases Prostate Cancer Risk: A Systematic Review and Meta-Analysis
Source: Nutrients. 2016 Oct 12;8(10):626. doi: 10.3390/nu8100626 (PMC5084014; doi:10.3390/nu8100626)
Supplement: Supplementary file 1 [file nutrients-08-00626-s001.docx]

Supplementary Materials: A Western Dietary Pattern Increases Prostate Cancer Risk: A Systematic Review and Meta-Analysis

Roberto Fabiani, Liliana Minelli, Gaia Bertarelli and Silvia Bacci

**Table S1.** Methodological quality of case-control studies included in the meta-analysis *.

| **First Author, Publication Year (Reference)** | **Adequate Definition of Cases** | **Representativeness of Cases** | **Selection of Control Subjects** | **Definition of Control Subjects** | **Control for Important Factor or Additional Factor †** | **Exposure Assessment** | **Same Method of Ascertainment for All Subjects** | **Non Response Rate ‡** | **Total Quality Scores** |
| --- | --- | --- | --- | --- | --- | --- | --- | --- | --- |
| Walker, 2005 [25] | 🟌 | 🟌 | --- | 🟌 | 🟌 | --- | 🟌 | --- | 5 |
| Ambrosini, 2008 [26] | 🟌 | 🟌 | 🟌 | 🟌 | 🟌🟌 | --- | 🟌 | 🟌 | 8 |
| De Stefani, 2009 [27] | 🟌 | 🟌 | --- | 🟌 | 🟌🟌 | 🟌 | 🟌 | 🟌 | 8 |
| Jackson, 2009 [28] | 🟌 | 🟌 | --- | --- | 🟌🟌 | 🟌 | 🟌 | --- | 6 |
| De Stefani, 2010 [29] | 🟌 | 🟌 | --- | 🟌 | 🟌🟌 | 🟌 | 🟌 | 🟌 | 8 |
| Jackson, 2013 [30] | 🟌 | 🟌 | --- | 🟌 | 🟌🟌 | 🟌 | 🟌 | --- | 7 |
| Rosato, 2014 [31] | 🟌 | 🟌 | --- | 🟌 | 🟌🟌 | 🟌 | 🟌 | 🟌 | 8 |
| Askari, 2014 [32] | 🟌 | 🟌 | --- | 🟌 | 🟌 | 🟌 | 🟌 | --- | 6 |
| Niclis, 2015 [33] | 🟌 | 🟌 | 🟌 | 🟌 | 🟌 | 🟌 | 🟌 | 🟌 | 8 |

* A study could be awarded a maximum of one star for each item except for the item Control for important factor or additional factor. † A maximum of 2 stars could be awarded for this item. Studies that controlled for age received one star, whereas studies that controlled for other important confounders (smoking and total energy intake/BMI) received an additional star. ‡ One star was assigned if there was no significant difference in the response rate between control subjects and cases by using the chi-square test (*p* < 0.05).

**Table S2.** Methodological quality of cohort studies included in the meta-analysis *.

| **First Author, Publication Year (Reference)** | **Representativeness of the Exposed Cohort** | **Selection of the Unexposed Cohort** | **Ascertainment of Exposure** | **Outcome of Interest Not Present at Start of Study** | **Control for Important Factor or Additional Factor †** | **Assessment of Outcome** | **Follow-Up Long Enough for Outcomes to Occur ‡** | **Adequacy of Follow-Up of Cohorts §** | **Total Quality Scores** |
| --- | --- | --- | --- | --- | --- | --- | --- | --- | --- |
| Tseng, 2004 [34] | 🟌 | 🟌 | 🟌 | 🟌 | 🟌🟌 | 🟌 | 🟌 | --- | 8 |
| Wu, 2006 [35] | 🟌 | 🟌 | 🟌 | 🟌 | 🟌🟌 | 🟌 | 🟌 | --- | 8 |
| Muller, 2009 [36] | 🟌 | 🟌 | 🟌 | 🟌 | 🟌🟌 | 🟌 | 🟌 | --- | 8 |

***** A study could be awarded a maximum of one star for each item except for the item Control for important factor or additional factor. † A maximum of 2 stars could be awarded for this item. Studies that controlled for age received one star, whereas studies that controlled for other important confounders (smoking and total energy intake/BMI) received an additional star. ‡ A cohort study with a follow-up time > 6 years was assigned one star. § A cohort study with a follow-up rate > 75% was assigned one star.


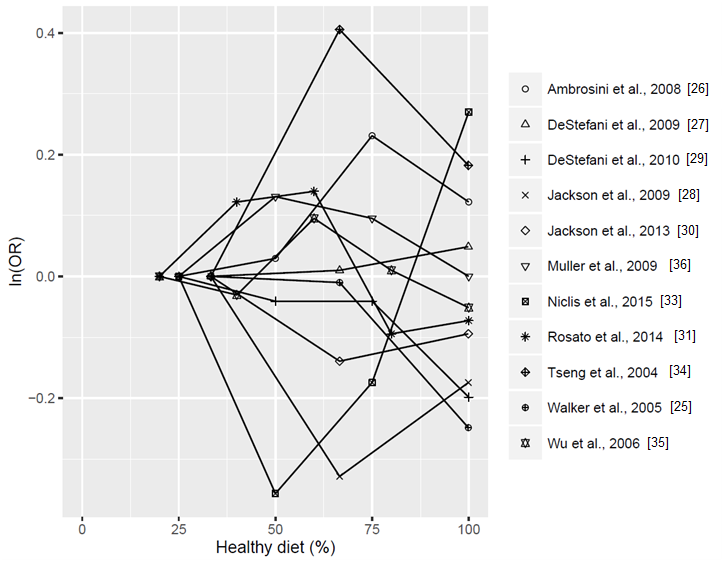


**Figure S1.** Dose-response plots of the relation between the intake of the “Healthy” dietary pattern and prostate cancer risk in the different studies included in the meta-analysis.

**
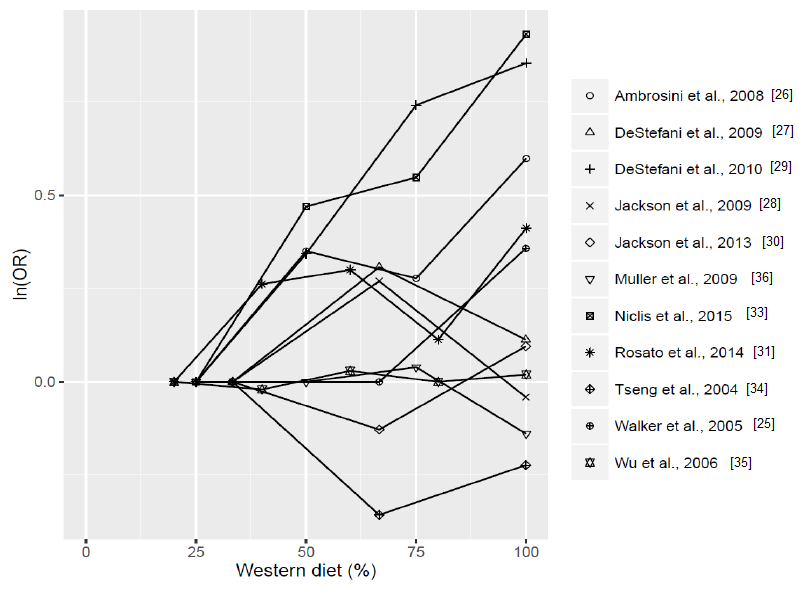
**

**Figure S2.** Dose-response plots of the relation between the intake of the “Western” dietary pattern and prostate cancer risk in the different studies included in the meta-analysis.


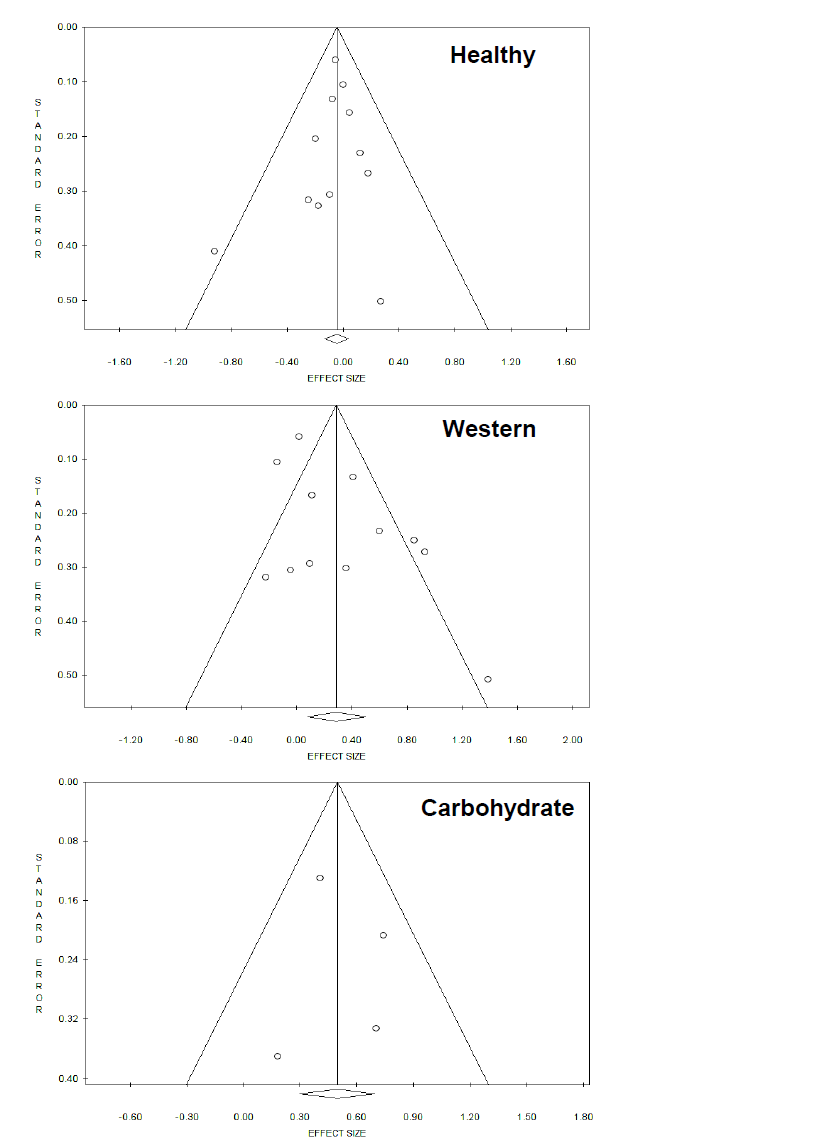


**Figure S3.** Funnel plots of studies included in the meta analysis evaluating the association between different dietary patterns (Healthy, Western and Carbohydrate) and prostate cancer risk.
